# Perceived mental effort correlates with changes in tonic arousal during attentional tasks

**DOI:** 10.1186/1744-9081-6-39

**Published:** 2010-07-08

**Authors:** Fleur M Howells, Dan J Stein, Vivienne A Russell

**Affiliations:** 1 Department of Psychiatry and Mental Health, Faculty of Health Sciences, University of Cape Town, Observatory, 7925, South Africa; 2 Department of Human Biology, Faculty of Health Sciences, University of Cape Town, Observatory, 7925, South Africa

## Abstract

**Background:**

It has been suggested that perceived mental effort reflects changes in arousal during tasks of attention. Such changes in arousal may be tonic or phasic, and may be mediated by the locus-coeruleus norepinephrine (LC-NE) system. We hypothesized that perceived mental effort during attentional tasks would correlate with tonic changes in cortical arousal, as assessed by relative electroencephalogram (EEG) band power and theta/beta ratio, and not with phasic changes in cortical arousal, assessed by P300 amplitude and latency.

**Methods:**

Forty-six healthy individuals completed tasks that engage the anterior and posterior attention networks (continuous performance task, go/no-go task, and cued target detection task). During completion of the three attentional tasks a continuous record of tonic and phasic arousal was taken. Cortical measures of arousal included frequency band power, theta/beta ratios over frontal and parietal cortices, and P300 amplitude and latency over parietal cortices. Peripheral measures of arousal included skin conductance responses, heart rate and heart rate variance. Participants reported their perceived mental effort during each of the three attentional tasks.

**Results:**

First, changes in arousal were seen from rest to completion of the three attentional tasks and between the attentional tasks. Changes seen between the attentional tasks being related to the task design and the attentional network activated. Second, perceived mental effort increased when demands of the task increased and correlated with left parietal beta band power during the three tasks of attention. Third, increased mental effort during the go/no-go task and the cued target detection task was inversely related to theta/beta ratios.

**Conclusion:**

These results indicate that perceived mental effort reflects tonic rather than phasic changes in arousal during tasks of attention. We suggest that perceived mental effort may reflect in part tonic activity of the LC-NE system in healthy individuals.

## Background

Arousal can be defined as a change in physiological and/or psychological responsiveness to internal or external stimuli. Early studies attributed changes in peripheral measures of sympathetic nervous system activity, such as skin conductance [1], to task-related changes in arousal [2,3]. According to the Yerkes and Dodson [4] theory, however, an individual who is underaroused or hyperaroused will perform a task poorly. This suggests that tonic levels of arousal need to be maintained within an optimal range in order to achieve successful completion of a task. In addition, the individual would then recruit the necessary phasic neural processes (also referred to as activation) for successful completion of the task [2,3]. Therefore poor performance during a task may relate to inappropriate tonic levels of arousal and/or phasic processes.

William James [5] defined attention as the "taking possession by the mind in clear and vivid form, of one out of what seem several simultaneously possible objects or trains of thought". Posner and Petersen [6] proposed two attentional networks that rely on interactions with arousal systems. (1) The anterior attentional network has been suggested to involve the detection of sensory targets and is strongly reliant on the anterior cingulate cortex. (2) The posterior attentional network has been suggested to involve sensory attention orienting and is reliant on the functioning of the posterior parietal cortex, superior colliculi and thalamic pulvinar nuclei [6]. Attention required for successful completion of a task requires an optimal level of arousal, which in turn requires activation of particular attentional network.

This work leads to the question of whether an individual would be able to report changes in arousal during performance of a cognitive task that required attention. Pribram and McGuinness [2] postulated that effort used during a voluntary mental process (such as attention) is related to the energy required to produce repeated changes in the "representational organization of information processing". Studies have shown that difficulty, complexity and stress-inducing tasks lead to increased subjective perceptions of mental effort which have in turn been related to increased physiological arousal as measured by increased skin conductance responses, heart rate, and heart rate variance [7-14]. Thus subjective perception of mental effort may reflect changes in arousal during performance of attentional tasks.

Arousal systems of the central nervous system arise from several nuclei in the reticular activating system of the brain stem [15]. These systems are classified according to their pathways and their specific neurotransmitters which include: (1) the locus coeruleus noradrenergic system (LC-NE), (2) the magnocellular basal forebrain/pedunculopontine cholinergic system, (3) the midbrain substantia nigra/ventral tegmental area dopaminergic system, (4) the dorsal raphe serotonergic system, and (5) the tuberomamillary hypothalamic histaminergic system [16].

The LC-NE system has been strongly related to arousal and attentional regulation [17-21]. Activation of the LC has been shown to increase cortical arousal (as measured by electroencephalographic (EEG) recordings) and improve the 'signal' (information that needs to be attended to) by decreasing the 'noise' (background non-relevant information) which has been related to tonic activity of the LC-NE neurons [22-24]. In addition the LC-NE system responds phasically to salient stimuli. This activity of the LC-NE system has been suggested to play an important role in the P300 component of an event-related potential (ERP) [25]. The P300 is a positive deflection of a stimulus-locked epoch in an EEG trace, such as is seen in an array of cognitive tasks [26]. The occurrence of the P300 has been suggested to reflect cortical updating [27], as is suggested by the 'orienting response' proposed by Sokolov [28,29]. This work indicates that individuals create a cortical representation of trials within a task. As the task endures and the individual completes more trials, the cortical representations of the trials are updated [28,29]. The extent of cortical updating has been shown to depend on the individual's level of arousal [30-32] and the value placed on the information being processed [33]. The role of tonic and phasic firing of the LC-NE system in the regulation of arousal during attention is not clear. As an improved 'signal-to-noise ratio' may serve to reflect the tonic changes in LC-NE system activity, so changes in information processing and cortical updating may serve to reflect the phasic changes in LC-NE system activity. At the same time it is not possible to disengage the tonic and phasic activities of the LC-NE system.

The aims of the present study were to determine: (1) whether changes in physiological levels of arousal increased during the completion of three voluntary attentional tasks and what aspects of the tasks were associated with these changes, (2) whether perceived mental effort reflected these changes in arousal during tasks of attention and what aspects of these tasks were associated with these changes and (3) whether perceived mental effort reflected tonic or phasic changes in arousal as observed in physiological recordings during the three tasks of attention.

To address the aims of the present study several physiological parameters were recorded during three voluntary attentional tasks. These parameters have previously been related to increased arousal and mental effort, and included: relative theta, alpha, and beta band power of frontal and parietal electrodes, amplitude and latency of parietal P300s, number and duration of skin conductance responses (SCRs), heart rate, and heart rate variance. Participants were asked to report their perceived mental effort during each of the three attentional tasks. In addition salivary cortisol measures were taken before and after the testing session to address the possible confounds of stress-related hypothalamic pituitary adrenal axis activation.

We hypothesized that perceived mental effort during attentional tasks would correlate with tonic changes in cortical arousal, as assessed by beta band power and theta/beta ratio in the EEG, and not with phasic changes in cortical arousal, assessed by P300 amplitude and latency. If perceived mental effort is related to tonic changes in arousal then this may reflect tonic activities of the LC-NE arousal system.

## Methods

### Participants

Forty-six healthy participants (28 females, 27.6 ± 5.3 years old) recruited from the postgraduate community of the University of Cape Town, South Africa, took part in the present study. Participation in the present study held no incentive and was voluntary. The study was approved by the Health Science Faculty Human Ethics Committee of the University of Cape Town, and the participants signed informed consent. The study was conducted in accordance with the Declaration of Helsinki [34]. Participants reported no psychiatric, psychological, substance use or dependence disorder, and did not have a current general medical condition or history of brain trauma. In addition participants had matriculated, with English as a first language.

### Experimental design

Participants were required to refrain from caffeine, cigarettes, alcohol, and non-prescription drugs for 18 hrs before testing. Testing of participants occurred between 09h30 and 13h30. Saliva for the cortisol assay was collected immediately before physiological recordings and immediately after (±1 hour apart). Physiological recordings were collected by a MP150 Biopac acquisition system (Biopac Systems Inc.) with amplifier modules set for EEG (electrodes of interest F_3_, F_4_, P_3_, P_4_), skin conductance responses, and electrocardiogram. The testing session comprised of five stages: (1) resting eyes open (2 min) and three attentional tasks: (2) a continuous performance task, (3) a go/no-go task, and (4) a cued target detection task. All of the above were programmed using E-prime 1.1. The physiological data and digital output from E-prime were collected on-line by Acqknowledge 3.8.1 software (Biopac Systems Incorporated). At least 10 min was permitted to ensure reliable signal conductance. The testing session was completed in a quiet unlit room to reduce distractors from the tasks at hand. The participants were tested only once and were naïve to the testing session. Thereafter the participants completed a visual analogue scale that expressed their perceived level of mental effort during performance of each of the attentional tasks. All data analyses were performed post-hoc to data acquisition.

### Continuous performance task repeated letter version

The continuous performance task involved three consecutive presentations of the letter 'S', the third 'S' was the target stimulus. Sixty-four trials were presented. The stimuli were present for 500 msec, with an interval of 100 msec. Non-target stimuli were presented for 500 msec in the centre of the computer screen with an interval of 100 msec. The non-target stimuli were letters of the alphabet which were not vowels.

### Go/no-go task

The go/no-go task required sustained attention in addition to response inhibition and delayed response to stimulus presentation. The visual go/no-go task used in the present study required the participant to respond to all consonants with the exception of the letter 'V'. No vowels were used. The 1^st ^condition of the go/no-go task was a go condition, in which all stimuli required responses by the participant as no Vs' were presented. The 2^nd ^condition of the go/no-go task was a no-go condition, in which participants needed to respond to the consonants that were non-Vs and inhibit their response on presentation of the Vs. The 3^rd ^condition of the go/no-go task was a go condition with a longer inter-stimulus interval of 3 500 msec unlike the inter-stimulus interval of 1 500 msec used in the 1^st ^and 2^nd ^condition. The 1^st ^and 2^nd ^conditions comprised 40 stimuli. In the 2^nd ^condition 20 of 40 stimuli were Vs which required response inhibition (50% target, 50% non-target split). The 3^rd ^condition comprised 20 stimuli. These three conditions were then repeated in reverse order forming a mirror image of the first three conditions.

### Cued target detection task

The cued target detection task used in the present study was an endogenous form of Posner's exogenous covert orienting task [35]. The cued target detection task required participants to maintain fixation on a central cue, a solid white circle in the centre of the computer screen. On either side of the central fixation cue was an outline of a rectangle that was grey in colour. The central fixation point and the grey rectangle outlines were present throughout the cognitive task. The participant was required to respond to the presentation of a square within either of the rectangles. For this task there were four conditions: (1) congruent cue and stimulus presentation; (2) incongruent cue and stimulus presentation; (3) double cue and stimulus presentation; and (4) no cue and stimulus presentation. Cues were presented for 500 msec and the stimulus was presented for 500 msec. The inter-stimulus interval was variable throughout the task, with durations of 500, 1000, or 1500 msec. The cued target detection task had 64 stimuli that were congruent; 16 stimuli that were incongruent; 16 stimuli that had double cueing; and 16 stimuli that had no cues. We only report event-related potential data from the congruent stimuli in the present paper.

### EEG relative band power analysis and P300 extraction

EEG data were collected with the use of EEG100C amplifier modules which were attached to the MP150 acquisition system (Biopac Systems Inc.), electrodes of interest were F_3_, F_4_, P_3_, and P_4_. Linked ear lobe reference was used. The EEG100C amplifier gain was set at 1000, mode normal, low pass filter set at 100 Hz, and high pass filter on at 0.1 Hz (application note 233, Biopac Systems Incorporated). The CAL1 input value was set at 10 with scale value set at 10 and the CAL2 input value was set at -10 with a scale value of -10. The sampling frequency of the software (Acqknowledge 3.8.1) was set at 500 Hz with units of recording set at μV. The EEG data was passed through a Hamming window, FIR band pass filter, low frequency at 0.05 Hz and high frequency at 30 Hz, number of coefficients was set at 4000, using Acqknowledge 3.8.1. The filtered EEG data for the different stages of the testing session were Fourier transformed extracting theta (θ, 4-7 Hz), alpha (α, 7-14 Hz), and beta (β, 15-30 Hz) frequency bands. The extracted absolute power was converted into relative band power (%).

Event-related potentials (ERPs) were extracted with Acqknowledge software (greater than +100 μV and less than -100 μV) as per digital inputs from E-prime for each of the three attentional tasks. The ERPs were baseline corrected for 100 msec before stimulus presentation and visually inspected. The window for the P300 component for the continuous performance task was 200-500 msec after the stimulus, for the go/no-go task (go and no-go stimuli) it was 250 - 500 msec, and for the cued target detection task (congruent trials only) it was 400-700 msec. The P300 amplitude was the point at which the height of the P300 was maximal. P300 latency was the time at which the P300 amplitude was maximal.

### Skin conductance responses

Skin conductance responses were measured with the GSR100C amplifier module which was attached to the MP150 acquisition system (Biopac Systems Inc.). The GSR100C module was set to measure phasic activity (AC), with the gain set at 10 μS/V, the low pass filter was set at 10 Hz and the high pass filter was set at 0.05 Hz, with a sampling frequency of 500 Hz. Scaling parameters on the software CAL1 input were set at 0. The CAL2 input value was set at 1 with a scale factor set at 10. Units of skin conductance were recorded in μS. A TSD203 skin conductance transducer was attached to the GSR100C amplifier module. The skin conductance data was passed through a Hamming window, FIR low pass filter of 1 Hz, and the number of coefficients was set at 2000, using Acqknowledge 3.8.1. The data were then analyzed for peaks exceeding a threshold value of 0.05 μS, the number of responses and their durations were extracted during each stage of the testing session and do not coincide with measures of task responses (see [36]).

### Heat rate and heart rate variance

Electrocardiograph data were collected with the use of three ECG100C amplifier modules which were attached to the MP150 acquisition system (Biopac Systems Inc.). The three ECG100C modules were connected to a TSD155C multi-lead ECG cable with built-in Wilson terminal (five leads). The ECG100C amplifier module's gain was set at 1000, mode normal, low pass filter set at 35 Hz, and high pass filter set at 0.5 Hz. The CAL1 input value was set at 10 with scale value set at 10. The CAL2 input value was set at -10 with scale value set at -10. The sampling frequency of the software (Acqknowledge 3.8.1) was set at 500 Hz with units of recording set at μvolts. The electrocardiogram data was passed through a Hamming window, FIR band pass filter, low frequency at 0.05 Hz and high frequency at 35 Hz, the number of coefficients was set at 4000, using Acqknowledge 3.8.1. Tachograms for the different stages of the testing session were obtained through a specialized software application, using Acqknowledge 3.9. The data then underwent a fully automated heart rate variance analysis (Biomedical Signal Analysis Software, Department of Applied Physics, University of Kuopio, Finland). Default parameters set in the software for autoregressive analysis were applied to the tachograms: low frequency 0.04 to 0.15 Hz and high frequency 0.15 to 0.4 Hz components. The autoregressive model order was set at 20. Heart rate, relative low frequency, relative high frequency, and the low to high frequency ratio were obtained from the data.

### Salivary cortisol assay

Salivettes^® ^(Sarstedt non-citric acid sterile cotton wool rolls) were used to collect saliva for the cortisol assay. Samples were stored at -80°C. Salimetrics LLC expanded range high sensitivity salivary cortisol enzyme immunoassay kits were used to determine cortisol concentrations. The assay procedure was carried out as recommended by the insert of the assay kits, Salimetrics Catalog No. 1-3002/1-3012, 96-Well Kit (lower detection limit 0.003 μg/dL else 0.083 nmol/L). All samples were analyzed in duplicate and the pH of all samples was within the range of accuracy for the enzyme immune-assay. Optical density was measured at 450 nm. Values were converted in the manner suggested in Salimetrics Catalog No. 1-3002/1-3012, 96-Well Kit using standards supplied with the kit.

### Visual analogue scale of perceived mental effort

The participants' perceived mental effort was assessed by means of a visual analogue scale [37]. On a single A4 landscape sheet there were three 10 cm vertical lines, one for each of the attentional tasks. At the top of the vertical line 'very high mental effort' was typed and at the bottom of the vertical line 'very little mental effort' was typed. Participants were asked to mark on each of the three vertical lines the amount of perceived mental effort they felt that they had used for each of the attentional tasks. The score (%) was the distance (cm) from the bottom of the vertical line.

### Statistical analysis

The Statistica 8 software package was used for the statistical analyses. Non-parametric statistics were used to analyze all data since the Shapiro-Wilks W test revealed that the data were not normally distributed. Wilcoxon matched pairs test was performed when comparing two dependent variables. Friedman ANOVA was performed for comparison of several dependent variables. Spearman's Rank R was used to determine correlations between variables. Figures report mean ± SEM. Bonferroni correction was applied to all data exceeding two comparisons (4 comparisons p < 0.0125, 3 comparisons p < 0.01667).

## Results

### Relative EEG band power and theta/beta ratio for each stage of the testing session

**Figure 1:**
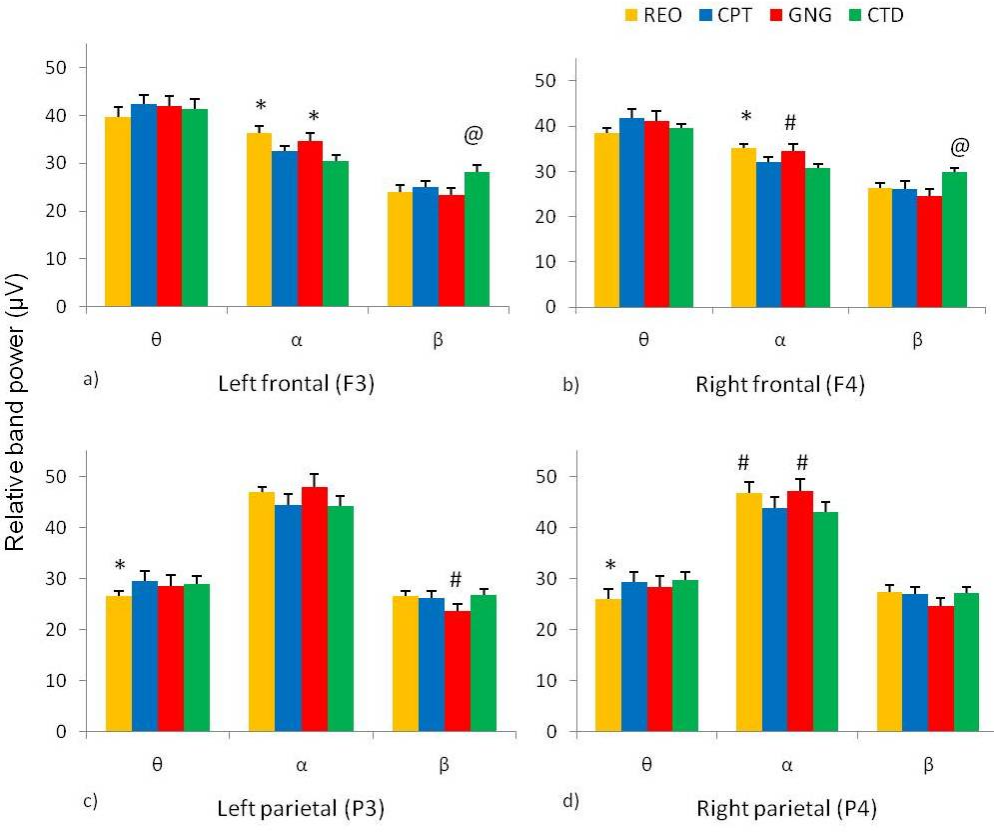
**Relative EEG band power during stages of the testing session**. Relative EEG band power during various stages of the testing session: resting eyes open (REO), continuous performance task (CPT), go/no-go task (GNG), and cued target detection task (CTD). Relative band power reported: theta (θ, 4-7 Hz), alpha (α, 7-14 Hz), and beta (β, 15-30 Hz) for frontal (F3 & F4) and parietal (P3 & P4) electrodes. a) *Left frontal (F3) α band power was higher during REO and GNG than CPT and CTD. ^@^β band power was higher during CTD than REO, CPT, and GNG. b) *Right frontal (F4) α band power was higher during REO than CPT and CTD. ^#^α band power was higher during GNG than CTD. ^@^β band power was higher during CTD than CPT and GNG. c) *Left parietal (P3) θ band power was lower during REO than CPT and CTD. ^#^β band power was lower during GNG than REO, CPT, and CTD. d) *Right parietal (P4) θ band power was lower during REO than CPT and CTD. ^#^α band power was higher during REO and GNG than during CTD (p < 0.0125, n = 46, mean ± SEM).

Friedman ANOVAs revealed differences in stages of the testing session (rest, continuous performance task, go/no-go task, and cued target detection task) for relative alpha and beta power for frontal electrodes (F3 relative α Chi Sqr_(3,46) _= 17.09, p < 0.001, F3 relative β Chi Sqr_(3,46) _= 26.17, p < 0.0001 and F4 relative α Chi Sqr_(3,46) _= 10.61, p = 0.014, F4 relative β Chi Sqr_(3,46) _= 19.48, p < 0.0002). Differences between the stages of the testing session were also revealed for relative theta and beta power for left parietal electrode (P3) and relative theta and alpha power for right parietal electrode (P4) (Figure 1). Wilcoxon matched pairs tests revealed the following differences. Left frontal electrode (F3) relative alpha band power was higher during the resting eyes open phase and the go/no-go tasks than during the continuous performance task and the cued target detection task. Left frontal electrode (F3) relative beta band power during the cued target detection task was higher than during resting eyes open, the continuous performance task, and the go/no-go task (Figure 1a). Right frontal electrode (F4) relative alpha band power was higher during the resting eyes open phase compared to continuous performance task and the cued target detection task. Relative alpha band power was higher during the go/no-go task than during the cued target detection task. Right frontal electrode (F4) relative beta band power was higher during the cued target detection task than during the continuous performance task and the go/no-go task (Figure 1b). Left parietal electrode (P3) relative theta band power was lower during resting eyes open than during the continuous performance task and the cued target detection task. Left parietal electrode (P3) relative beta band power was lower during the go/no-go task than during resting eyes open, the continuous performance task, and the cued target detection task (Figure 1c). Right parietal electrode (P4) relative theta band power was lower during resting eyes open than during the continuous performance task and the cued target detection task. Right parietal electrode (P4) relative alpha band power during resting eyes open and the go/no-go task were higher than during the cued target detection task (Figure 1d).

**Figure 2:**
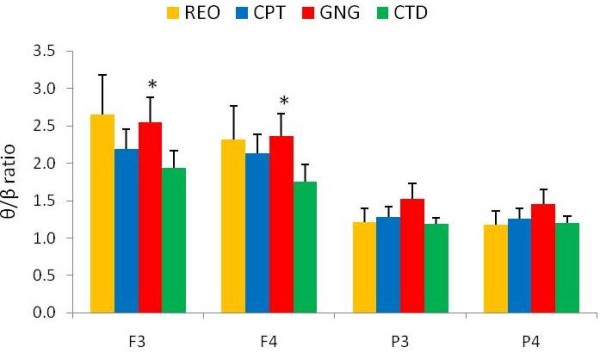
**Relative theta/beta (θ/β) ratio for frontal (F3 & F4) and parietal (P3 & P4) electrodes for the stages of the testing session**. Relative theta/beta (θ/β) ratio for frontal (F3 & F4) and parietal (P3 & P4) electrodes for the various stages of the testing session: resting eyes open (REO), continuous performance task (CPT), go/no-go task (GNG), and cued target detection task (CTD). *θ/β ratio for F3 and F4 was greater during GNG than during CTD (p < 0.0125, n = 46, mean ± SEM).

Friedman ANOVA revealed differences between the three attentional tasks at frontal electrodes (F3 & F4) for relative theta/beta ratios (F3 θ/β ratio Chi Sqr_(3,46) _= 12.55, p < 0.01) and F4 θ/β ratio Chi Sqr_(3,46) _= 8.23, p < 0.05). Wilcoxon matched pairs test for left frontal electrode (F3) revealed that the relative theta/beta ratio was higher during the go/no-go task than the cued target detection task. Wilcoxon matched pairs test for the right frontal electrode (F4) revealed that the theta/beta ratio was higher during the go/no-go task than the cued target detection task (Figure 2).

### Parietal P300 latency and amplitude for the three attentional tasks

**Figure 3:**
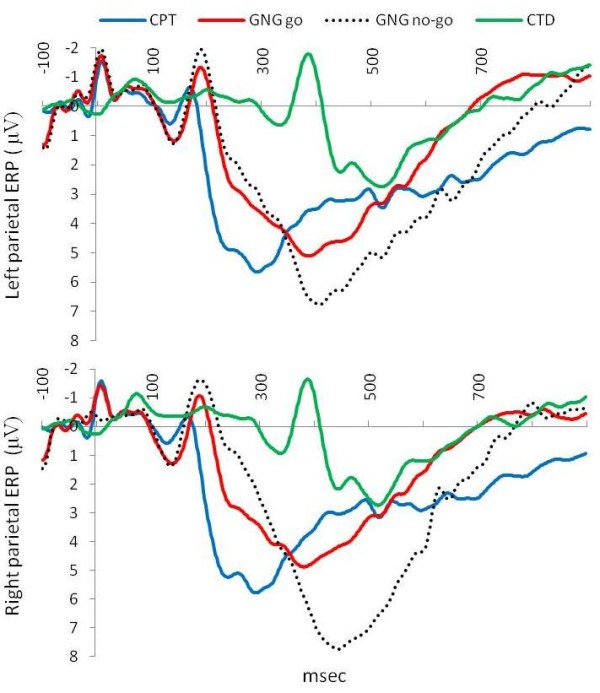
**Grand mean event-related potentials (ERPs) for parietal electrodes (P3 and P4) during the three attentional tasks**. Grand mean event-related potentials (ERPs) for parietal electrodes (P3 and P4) during the three attentional tasks: continuous performance task (CPT), go/no-go task (go and no-go conditions; GNG), and cued target detection task (CTD).

**Figure 4:**
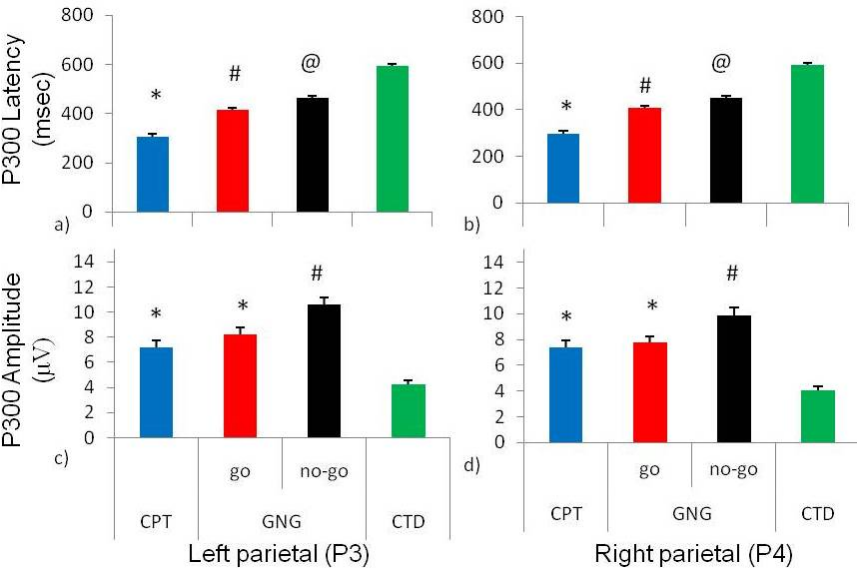
**Latency and amplitude of the event-related potentials (ERPs) of parietal electrodes (P3 & P4) during the three attentional tasks**. Latency and amplitude of the event-related potentials (ERPs) of parietal electrodes (P3 & P4) during the three attentional tasks: continuous performance task (CPT), go/no-go task (go and no-go conditions; GNG), and cued target detection task (CTD). a) For left parietal P300 latency the *CPT was shorter than GNG and CTD, ^#^GNG go condition P300 latency was shorter than GNG no-go condition and CTD ^@^GNG no-go condition P300 latency was shorter than CTD b) For right parietal P300 latency the *CPT was shorter than GNG and CTD, ^#^GNG go condition P300 latency was shorter than GNG no-go condition and CTD. ^@^GNG no-go condition P300 latency was shorter than CTD. c) For left parietal P300 amplitude the *CPT and GNG go condition were smaller than GNG no-go condition and greater than CTD. The ^#^GNG no-go condition P300 amplitude was greater than CTD. d) The right parietal P300 amplitude *CPT and GNG go condition was smaller than GNG no-go condition and was greater than CTD. The ^#^GNG no-go condition P300 amplitude was greater than CTD.

Friedman ANOVAs revealed differences in the three attentional tasks for P300 latency and amplitude for left and right parietal electrodes and during the no-go condition of the go/no-go task (P3 P300 latency Chi Sqr_(3,46) _= 111.4, p < 0.0001, P4 P300 latency Chi Sqr_(3,46) _= 114.5, p < 0.0001, P3 P300 amplitude Chi Sqr_(3,46) _= 66.15, p < 0.0001 and P4 P300 amplitude Chi Sqr_(3,46) _= 66.15, p < 0.005). Wilcoxon matched pairs tests revealed the following: left parietal (P3) electrode P300 latency during the continuous performance task was shorter than the go/no-go task and cued target detection task. The grand mean event-related potentials (ERPs) for parietal electrodes, P3 and P4, are shown in (Figure 3). The P300 latency during the go condition of the go/no-go task was shorter than during the no-go condition and both were shorter than P300 latency during the cued target detection task (Figure 4a). Right parietal (P4) electrode P300 latency for the continuous performance task was shorter than the go/no-go task and the cued target detection task. The go/no-go go condition had shorter latency than the go/no-go no-go condition and the cued target detection task while the go/no-go no-go condition had shorter P300 latency than the cued target detection task (Figure 4b). Left parietal (P3) electrode P300 amplitude for the continuous performance task and the go/no-go go condition was lower than the go/no-go no-go condition and higher than the cued target detection task. The go/no-go no-go condition had higher P300 amplitude than the cued target detection task (Figure 4c). Right parietal (P4) electrode P300 amplitude for the continuous performance task and the go/no-go go condition was lower than the go/no-go no-go condition and higher than the cued target detection task. The go/no-go no-go condition had higher P300 amplitude than the cued target detection task (Figure 4d).

### Response times for each of the three attentional tasks and their conditions

Friedman's ANOVA revealed significant differences in response times between tasks (Chi Sqr_(2,46) _= 44.74, p < 0.0001). The Wilcoxon matched pairs test revealed shorter response times for the continuous performance task (386 ± 85 msec) compared to the go/no-go task (418 ± 21 msec) and cued target detection task (473 ± 49 msec). In addition response times for the go/no-go task were shorter than the cued target detection task. During the go/no-go task, response inhibition errors were recorded, with an average of 2.3 ± 0.25 errors made during the task.

### Skin conductance responses and durations

**Figure 5:**
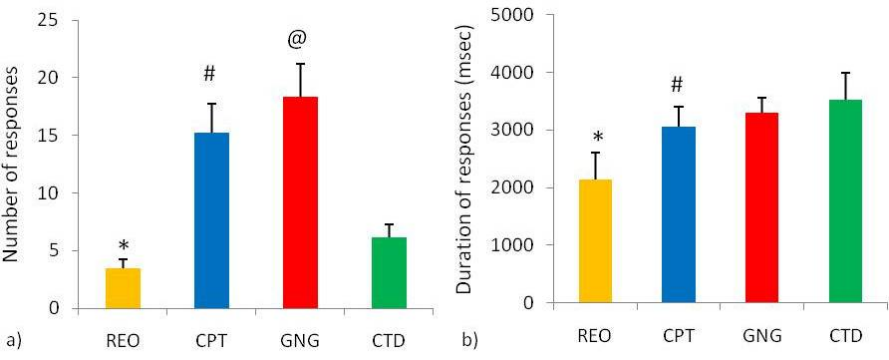
**Skin conductance responses and their duration during stages of the testing session**. a) Skin conductance responses recorded during resting eyes open (REO), continuous performance task (CPT), go/no-go task (GNG), and cued target detection task (CTD). *The number of skin conductance responses during REO were fewer than at all other stages. ^#^CPT responses were fewer than responses made during the GNG and greater than responses made during the CTD. ^@^GNG responses were greater than during the CTD. b) Duration of skin conductance responses. *The duration of responses during REO was shorter than at all other stages. ^#^CPT duration of response was shorter than the duration of responses during the GNG and CTD (p < 0.0125, n = 46, mean ± SEM).

Friedman ANOVA revealed differences in the number of skin conductance responses (Chi Sqr_(3,46) _= 92.93, p < 0.0001) and duration of response (Chi Sqr_(3,46) _= 24.67, p < 0.0001) during the different stages of the testing session (Figure 5).

Wilcoxon matched pairs test revealed that the number of skin conductance responses was fewer during resting eyes open than during the three attentional tasks. Fewer skin conductance responses were made during the continuous performance task than during the go/no-go task and the cued target detection task. A greater number of skin conductance responses were made during the go/no-go task than during the cued target detection task (Figure 5a).

Wilcoxon matched pairs test revealed that the duration of skin conductance responses was shorter during resting eyes open than during the three attentional tasks. Additionally the skin conductance responses were shorter during the continuous performance task compared to the go/no-go task and the cued target detection task (Figure 5b).

### Electrocardiogram heart rate and heart rate variance for each stage of the testing session

**Figure 6:**
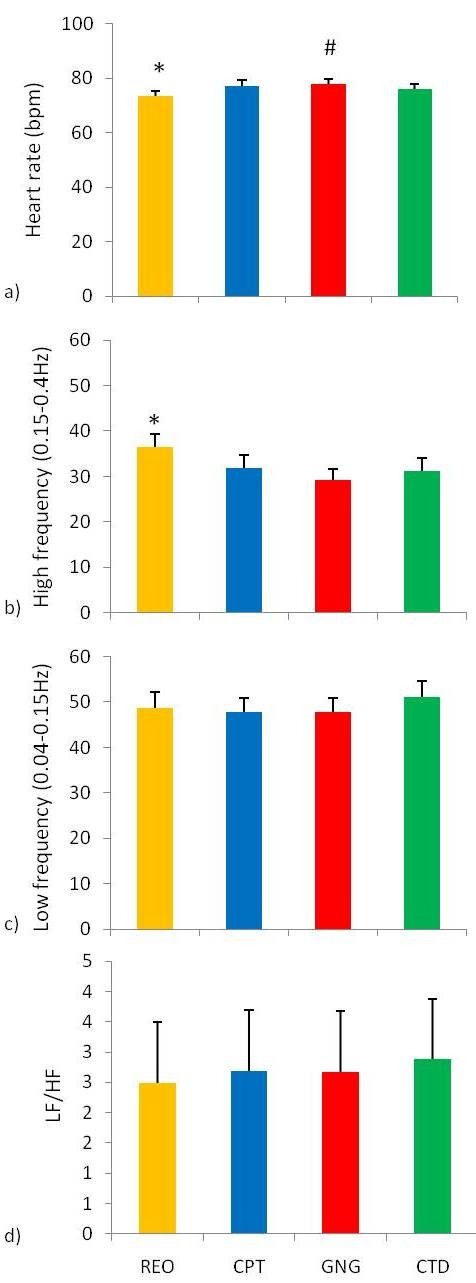
**Heart rate and heart rate variance parameters during stages of the testing session**. a) Heart rate and heart rate variance parameters during: resting eyes open (REO), continuous performance task (CPT), go/no-go task (GNG), and cued target detection task (CTD). *Heart rate during REO was lower than during CPT, GNG, and CTD tasks (p < 0.0125, n = 46, mean ± SEM). ^#^Heart rate during GNG was higher than during the CTD task (p < 0.0125, n = 46, mean ± SEM), b) *The high frequency range during REO was greater than during the GNG task (p < 0.0125, n = 46, mean ± SEM). c) No differences were found in the low frequency range. d) No differences were found in the low frequency/high frequency ratio of heart rate variance parameters.

Friedman ANOVA revealed differences in heart rate during the different stages of the testing session (Chi Sqr_(3,46) _= 49.89, p < 0.0001) and differences in high frequency heart rate variance (Chi Sqr_(3,46) _= 9.77, p < 0.05; Figure 6).

Wilcoxon matched pairs test revealed heart rate was lower during resting eyes opened compared to all other stages of the testing session. In addition, heart rate was higher during the go/no-go task than during the cued target detection task (Figure 6a). Wilcoxon matched pairs test revealed high frequency heart rate variance was higher during resting eyes open than during the go/no-go task (Figure 6b). No differences were found in low frequency heart rate variance parameters (Figure 6c) or the low frequency/high frequency ratio (Figure 6d).

### Salivary cortisol before and after the testing session

The Wilcoxon matched pairs test revealed no significant difference between salivary cortisol levels before (0.16 ± 0.1 μg/dL) and after testing (0.17 ± 0.13 μg/dL). In addition no significant effects were found for gender, or use of contraceptive pill.

### Perceived mental effort during the three attentional tasks

**Figure 7:**
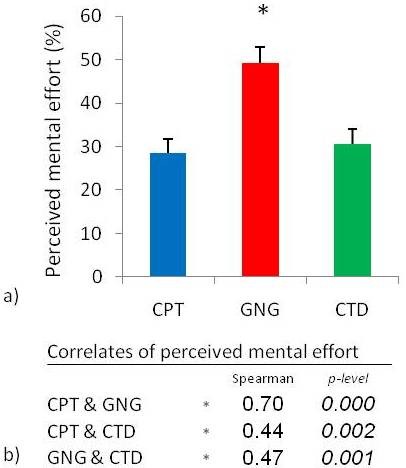
**Perceived mental effort during the three attentional tasks**. a) Perceived mental effort during the three attentional tasks: continuous performance task (CPT), go/no-go task (GNG), and cued target detection task (CTD). *Perceived mental effort was higher during the GNG task than during the CPT and the CTD. b) Strong positive correlations were observed between perceived mental effort during the three attentional tasks (p < 0.01667, n = 46, mean ± SEM).

Friedman ANOVA revealed differences in perceived mental effort during performance of the three attentional tasks (Chi Sqr_(2,46) _= 30.83, p < 0.0001) (Figure 7a). Wilcoxon matched pairs test revealed that greater mental effort was required when completing the go/no-go task than during the continuous performance task and the cued target detection task. Spearman rank order correlation showed strong relationships between perceived mental effort reported during each of the three attentional tasks (Figure 7b).

### Physiological correlates of perceived mental effort during the three attentional tasks

Spearman's rank order correlation analysis was performed to determine relationships between perceived mental effort and physiological measures recorded during completion of the attentional tasks (Table. 1).

**Table 1:** Relationships between perceived mental effort and physiological parameters measured during the three attentional tasks.

		Continuous Performance Task			Go/No-Go Task			Cued Target Detection Task		
		Spearman		*p-value*	Spearman		*p-value*	Spearman		*p-value*
*Left frontal (F3)*	*relative θ (4-7 Hz) power*		-0.09	*0.533*	*	-0.37	*0.011*	**	-0.33	*0.024*
	*relative α (7-13 Hz) power*		-0.08	*0.588*		0.21	*0.16*		0.11	*0.47*
	*relative β (13-30 Hz) power*		0.08	*0.59*		0.24	*0.115*	*	0.39	*0.008*
	*θ/β ratio*		-0.11	*0.461*	**	-0.31	*0.035*	*	-0.37	*0.011*
*Right frontal (F4)*	*relative θ (4-7 Hz) power*		0.11	*0.454*		-0.21	*0.166*	**	-0.34	*0.021*
	*relative α (7-13 Hz) power*		-0.1	*0.523*		0.1	*0.522*		-0.02	*0.912*
	*relative β (13-30 Hz) power*		-0.06	*0.696*		0.25	*0.092*	*	0.48	*0.001*
	*θ/β ratio*		0.06	*0.699*		-0.27	*0.074*	*	-0.43	*0.003*
*Left parietal (P3)*	*relative θ (4-7 Hz) power*		0.04	*0.795*		-0.16	*0.275*		-0.18	*0.232*
	*relative α (7-13 Hz) power*		-0.27	*0.065*		0.01	*0.949*		-0.03	*0.819*
	*relative β (13-30 Hz) power*	**	0.29	*0.047*	*	0.38	*0.009*	*	0.4	*0.006*
	*θ/β ratio*		-0.18	*0.237*	*	-0.37	*0.011*	*	-0.38	*0.009*
*Right parietal (P4)*	*relative θ (4-7 Hz) power*		0.01	*0.936*		-0.17	*0.258*		-0.23	*0.126*
	*relative α (7-13 Hz) power*		-0.22	*0.143*		-0.01	*0.944*		0.13	*0.399*
	*relative β (13-30 Hz) power*		0.18	*0.221*	*	0.37	*0.01*		0.21	*0.157*
	*θ/β ratio*		-0.14	*0.35*	*	-0.36	*0.015*		-0.25	*0.097*
										
P300 latency	*Left parietal (P3)*		-0.04	*0.776*		-0.13	*0.403*		0.01	*0.966*
	*Right parietal (P4)*		-0.1	*0.504*		-0.09	*0.564*		-0.08	*0.579*
	*Left parietal (P3) for no-go trials*					0.01	*0.939*			
	*Right parietal (P4) for no-go trials*					0.08	*0.616*			
										
P300 amplitude	*Left parietal (P3)*		0.12	*0.422*		-0.02	*0.886*		-0.05	*0.725*
	*Right parietal (P4)*		0.16	*0.286*		0.02	*0.916*		-0.05	*0.753*
	*Left parietal (P3) for no-go trials*					0.13	*0.395*			
	*Right parietal (P4) for no-go trials*					0.02	*0.88*			

Response time			-0.04	*0.779*		0.04	*0.773*		-0.07	*0.624*
Errors of inhibition made during no-go conditions						0.11	*0.478*			
										
Number of skin conductance responses			-0.17	*0.249*		-0.02	*0.907*		0.01	*0.946*
Duration of skin conductance responses			-0.13	*0.387*		-0.01	*0.947*		-0.12	*0.436*
										
Heart Rate			-0.08	*0.608*		0.11	*0.454*		0.01	*0.972*
Heart rate variance low frequency (0.04-0.15 Hz)			0.12	*0.447*		0.26	*0.085*		0.17	*0.248*
Heart rate variance high frequency(0.15-0.4 Hz)			0.03	*0.819*	**	-0.32	*0.03*		-0.24	*0.101*
Heart rate variance LF/HF			0.07	*0.647*	**	0.34	*0.022*		0.25	*0.09*

Spearman's rank order correlations between physiological parameters and perceived mental effort during each of the three attentional tasks: continuous performance task, go/no-go task, and the cued target detection task (** p < 0.05, * p < 0.01667, n = 46).

Perceived mental effort correlated positively with left parietal beta (P3) band power during the three tasks of attention. Right parietal beta band power during the go/no-go task also increased with increased perceived mental effort. Perceived mental effort correlated negatively with relative theta/beta ratios during the go/no-go task, for left frontal (F3) and parietal (P3 & P4) electrodes, during the cued target detection task a similar correlation was found for the left frontal (F3) and left parietal (P3) electrodes. Perceived mental effort correlated positively with relative theta during the go/no-go task and cued target detection task, for left frontal (F3) electrode, in addition during the cued target detection task a similar correlation was found for the right frontal (F4) electrode. Perceived mental effort during the go/no-go task correlated negatively with heart rate variance high frequency band and positively with the heart rate variance low frequency band and high frequency band ratio.

No relationships between perceived mental effort during the three attentional tasks were found for response times, errors of inhibition, skin conductance measures, or for P300 latency and amplitude (Table. 1).

## Discussion

The main findings were as follows: First, changes in arousal were seen from rest to completion of the three attentional tasks and between the attentional tasks. Changes seen between the attentional tasks being related to the task design and the attentional network activated. Second, perceived mental effort increased when demands of the task increased and correlated with left parietal beta band power during the three tasks of attention. Third, increased mental effort during the go/no-go task and the cued target detection task was inversely related to theta/beta ratios.

First, several changes in arousal were seen from rest to completion of the three attentional tasks. Cortical relative alpha band power was higher during rest and during the go/no-go task than during the continuous performance task and cued target detection task. The continuous performance task and cued target detection task required the individual to keep information in mind (cues) preceding the presentation of the target stimulus. Decreased alpha band power is thought to be a measure of increased attentional performance [38], and so may be related to cues being presented in the continuous performance task and cued target detection task. Given that alpha band power decreases with increased mental load [11,39] and increases with anticipatory waiting during attentional tasks [40] or during increased focused attention and/or increased arousal [41,42], the present findings suggest that during the continuous performance task and the cued target detection task there was increased mental load due to cueing systems of the tasks. While the increased alpha band power during the go/no-go task may reflect the lack of cueing and anticipation of the other two tasks.

Relative beta band power during the cued target detection task was higher over frontal cortical electrodes than during the continuous performance task and go/no-go task. Increased beta band power over the frontal cortex has been associated with deficits in sustained attentional performance [43,44]. However this increase may simply suggest that during the cued target detection task there was disengagement of the anterior attentional network, given that the reflexive cued target detection task would require increased activation of the posterior attentional network [6,19]. This hypothesis is supported by the fact that frontal beta band power was further reduced during the go/no-go task which required increased activation of the anterior attentional network. In addition covert endogenous shifting of attention that is necessary during the cued target detection has been correlated with frontal eye field 18-34 Hz oscillations [45], consistent with the present findings.

During the go/no-go task theta/beta ratios were higher over the frontal areas than during the continuous performance task and cued target detection task. This finding is of interest as individuals with ADHD are known for their poor attentional performance, which is accentuated in tasks that require response inhibition such as the go/no-go task. It has been suggested that individuals with ADHD are incapable of increasing their arousal levels and this is reflected in high theta/beta ratios [46]. The present study suggests that healthy individuals increased their theta/beta ratio when increased arousal was required, during the go/no-go task. This finding supports Barry and colleagues' suggestion that the theta/beta ratio is not a good physiological measure of the suggested hypo-arousal seen in individuals with ADHD [47,48]. In addition activation of working memory during the continuous performance task and the cued target detection task due to cueing stimuli may account for increased theta band power relative to beta band power over the frontal cortex [49].

P300 amplitudes and latencies were extracted from parietal electrode sites as these have been suggested to be maximal over the parietal cortices during attentional tasks [30] and may serve to reflect in part the phasic activities of the LC-NE system [25]. Bilaterally P300 latencies were shorter for the continuous performance task when compared to the go/no-go task (go and no-go conditions) and the cued target detection task. In addition left parietal P300 amplitudes were lower during the continuous performance task compared to the go/no-go (go and no-go conditions) task and the cued target detection task. It has been suggested that the P300 is a representation of the neural processing required for cortical updating [27]. The 'orienting response' suggests that an individual creates a cortical representation of trials within a task, as the task endures and the individual completes more trials, the cortical representations of the trials are continuously updated [28,29]. The extent of this cortical updating or 'orienting response' is dependent on the individual's state of arousal and may be affected by changes of tonic arousal levels and phasic arousal activity [30-32]. In addition several factors have been shown to affect the latency and amplitude of the P300. (1) Habituation to task and repetition of task, as shown in an oddball task that led to decrements in P300 amplitude sequentially for each of the ten times the task was repeated [50]. (2) Task difficulty as shown in a series of mathematical tasks that gradually increased their level of difficultly, the P300 amplitudes formed a U-shaped trend from 'extremely easy' to 'extremely difficult' [51].

The present findings support these studies. To complete the continuous performance task the individual was required to respond to the presentation of a third consecutive 'S', the reduced latency and reduced amplitude may relate to the reduced cortical updating required as the individual was primed by the first and the second 'S'. To complete the cued target detection task the individual received a cue in their peripheral visual field and responded to the target stimulus that was also in their peripheral visual field. The increased latency and reduced amplitude may reflect the exogenous orienting required that then effected the cortical updating of the peripheral images. To complete the go/no-go task the individual received no cueing system, the increased latency and increased amplitude relative to the continuous performance task may reflect an increase in cortical updating due to the 'no-cueing' design of the task. In addition during the no-go trials the P300 amplitude and latency were greater than during the go trials of the go/no-go task. The no-go trials required individuals to inhibit their response that would require increased cortical updating to prevent error responses.

Phasic skin conductance responses increased while individuals completed the three attentional tasks, reflected in both the number and the duration of responses when compared to resting conditions. Phasic skin conductance has previously been related to phasic changes in sympathetic arousal when orienting toward novel or salient stimuli [28,30]. The increased phasic skin conductance during completion of the three attentional tasks suggests increased sympathetic arousal [36] and can be related to activation of the phasic orienting reflex [52]. A parameter of heart rate variance, high frequency activity, decreased upon completion of the three attentional tasks. Decreased heart rate variance in the high frequency range suggests that there was a decrease in parasympathetic control of heart activity. This is supported by the increase in heart rate, which would occur with decreased parasympathetic control that would increase the impact of the sympathetic activity. However, activation of the 'stress response' did not occur as there was no difference in cortisol measures taken before and after the testing session.

Comparison of phasic skin conductance changes between tasks showed that during the go/no-go task individuals made a greater number of skin conductance responses (and increased heart rate) relative to the continuous performance task and cued target detection task. This suggests that there was a further increase in arousal during the go/no-go task over and above that which was seen during the continuous performance task and cued target detection task. These differences may have resulted from several additional challenges in the go/no-go task. Firstly the go/no-go task did not have a cueing system. Therefore, the participants were not primed to the presentation of the stimulus as occurred during the other two tasks. Secondly two of the go/no-go task conditions required either inhibition of response or delay of response. We suggest that these factors increased peripheral arousal during the go/no-go task.

Phasic increases in skin conductance during the cued target detection task displayed a different pattern compared to the continuous performance task and the go/no-go task. The cued target detection task led to fewer skin conductance responses and the duration of the responses were longer than during the other two tasks. The cued target detection task was the only task that involved different interstimulus intervals from trial to trial (500 msec or 1000 msec or 1500 msec). Previously it has been shown that increasing the interstimulus interval results in increased amplitude of phasic skin conductance responses [53] which may account for the present finding. In addition we suggest that this effect may be a result of the peripheral cue and stimulus processing and the additional covert orienting and reflexive nature of the cued target detection task.

Secondly, difficulty, complexity and stress induction during tasks has previously been reported as perceived mental effort which was related to changes in physiological arousal [7,8,10-12,54]. From these studies it has also been suggested that perceived mental effort may result from a balance between tonic levels of arousal and phasic LC-NE activity [30]. Perceived mental effort was found to be higher during the go/no-go task than during the continuous performance task and the cued target detection task. The go/no-go task was the second attentional task that was performed, which suggests that novelty of the testing environment did not play a role in the increased perceived mental effort reported for the go/no-go task, therefore not related to depletion of mental resources. The go/no-go task was different in that response inhibition and delays in responding were required for two of the go/no-go task conditions. In addition there was no cueing system in the go/no-go task which did occur for both the continuous performance task and the cued target detection task. Physiologically completion of the go/no-go task showed increased arousal levels including the number of skin conductance responses, heart rate, and relative alpha band power. This suggests that mental effort is related to increased arousal during the go/no-go task.

There were several cortical arousal measures that were related to perceived mental effort, one relationship was found across all three of the attentional tasks, being left parietal relative beta band power. The greater the perceived mental effort, the greater the left parietal relative beta band power was. This suggests that increased left parietal relative beta band power may reflect increased arousal levels required to maintain attention during attentional tasks. Consistent with this hypothesis, individuals that have been suggested to be unable to increase their arousal, ADHD, show decreased beta band power and increased levels of effort than controls [47,48,55].

Third, relative theta/beta ratios were inversely related to perceived mental effort during the go/no-go task and the cued target detection task. Earlier we suggested that increased theta/beta ratios were related with increased arousal, due to several aspects of the go/no-go task. The present finding suggests that with increased mental effort an individual's ability to maintain an optimal level of arousal decreases, during the go/no-go task. However during the cued target detection task which showed similar levels of mental effort as the continuous performance task these relationships may relate to the reflexive nature of the task. Importantly the pattern of theta band power, beta band power, and theta/beta ratio is different for the go/no-go task and the cued target detection task. The go/no-go task showed stronger correlates with posterior cortices (parietal) while the cued target detection task showed stronger correlates with anterior cortices (frontal). Earlier we noted that the go/no-go task employs the anterior attentional network, while the cued target detection task employs the posterior attentional network [6]. Finding this differential pattern related with mental effort suggests that brain areas that are not being directly activated for a cognitive task may be held in a tonic state ready for activation if required.

Finally, the only peripheral arousal measure that was related to perceived mental effort occurred during the go/no-go task. This included two of the reported parameters of heart rate variance, revealing an inverse relationship with high frequency activity and a positive relationship with low to high frequency ratio. The go/no-go task showed higher arousal levels when compared to the other two attentional tasks. This suggests that there was a decrease in parasympathetic input during the go/no-go task and may be related to increased tonic arousal levels due to the requirements of the task as discussed earlier.

## Limitations

The present study has several limitations. First, scalp electrodes that were used to record of EEG signals represent an average of multiple surface field potentials, and cannot be localized to any particular area of the brain. Thus, although the theoretical implications regarding the role of the LC-NE system are based in the animal literature, any more detailed anatomical arguments remain speculative. Second, no record of skin conductance levels, a measure of peripheral tonic arousal, was taken. Thus, we are not able to directly determine whether mental effort has a relationship to peripheral tonic arousal. Third, the present study only included 'control' participants, future studies would include disorders where arousal and attention deficits have been implicated, such as ADHD and several other psychiatric disorders.

## Conclusions

Perceived mental effort during attentional tasks correlated with tonic changes in cortical arousal, as assessed by left parietal beta band power and theta/beta ratio in the EEG, and not with phasic changes in cortical arousal, assessed by P300 amplitude and latency and skin conductance responses. Release of NE has been shown to increase cortical arousal and improve the 'signal' by decreasing the 'noise' which has been related to tonic activity of the LC nucleus [22-24]. We suggest that perceived mental effort may reflect in part the tonic activity of the LC-NE system in healthy individuals.

## List of abbreviations used

ADHD: Attention-deficit/hyperactivity disorder; CPT: continuous performance task; CTD: cued target detection task; EEG: electroencephalogram; GNG: go/no-go task; LC: locus coeruleus; NE: norepinephrine

## Competing interests

The authors declare that they have no competing interests.

## Authors' contributions

FMH conducted the research, analyse, and is the primary author of this work. DJS provided input to the manuscript. VAR supervised the study and provided input to the manuscript. All authors read and approved the final manuscript.
